# Bridging community and facility care: cost-effectiveness of implementing 2019 childhood pneumonia management guidelines in India

**DOI:** 10.1186/s12962-026-00792-3

**Published:** 2026-07-02

**Authors:** Archana Thakur, K. P. Junaid, Devang R. Raval, Dhaval Parmar

**Affiliations:** HTA Resource Hub, Indian Institute of Public Health Gandhinagar (IIPHG), Gandhinagar, Gujarat India

**Keywords:** Cost-effectiveness, Childhood pneumonia, Treatment, Economic evaluation, Decision analytic model

## Abstract

**Objective:**

The present study evaluates the cost-effectiveness of India’s 2019 childhood pneumonia management guidelines compared with no treatment, assuming 100% coverage, from a health system perspective.

**Methodology:**

The cost-effectiveness analysis parameterised a decision tree model in Microsoft Excel to estimate the incremental cost and effectiveness. Costing followed the normative approach, guided by national guidelines and the Indian Public Health Standards (IPHS) 2022, to define the types and quantities of resources. Published literature informed epidemiological inputs. Health effects were estimated as disability-adjusted life years (DALY) averted, using the World Health Organisation (WHO) DALY calculation template. The analysis discounted future health outcomes and capital costs at 3% to account for time preference. We reported our results as incremental cost per DALY averted. A probability sensitivity analysis in R software was conducted to estimate the joint effect of parameter uncertainties.

**Results:**

Treating fast-breathing pneumonia in accordance with the 2019 national childhood pneumonia management guideline incurs an incremental cost of 7.83 USD per DALY averted. For possible serious bacterial infections (PSBI), the cost-effectiveness varies by severity and treatment regimen. When managed in an outpatient setting, the additional cost per DALY averted is 2.1 USD, whereas treating PSBI in an inpatient (standard) setting increases the additional cost per DALY averted to 11.6 USD.

**Conclusion:**

India’s national childhood pneumonia management guidelines are highly cost-effective and should be scaled up nationwide to reduce the pneumonia burden and improve child survival.

**Supplementary Information:**

The online version contains supplementary material available at https://doi.org/10.1186/s12962-026-00792-3.

## Background

Every minute, a child dies in India [1]. Under-five mortality rates (U5MR) have declined significantly from 127 per 1000 live births in 1990 to 29 per 1000 live births in 2023; however, the urban-rural gap still persists, with U5MR of 33/1000 live births in rural areas and 20/1000 in urban areas [2]. Although urban statistics on child mortality are not disaggregated to reflect the burden among the urban poor, studies have reported a higher burden in these populations [3, 4]. This highlights the gaps in access to healthcare services in rural communities and urban slums, particularly in addressing major causes of childhood illnesses.

With 28 million cases and 0.14 million deaths, pneumonia is the leading cause of childhood mortality after perinatal causes, i.e., prematurity, low birth weight (LBW) & birth asphyxia; it accounts for 17% of total child deaths in India [5, 6]. This underscores the importance of prioritising resources to address childhood pneumonia and of adopting and scaling up effective interventions, particularly in rural areas, thereby increasing coverage.

In India, combating childhood pneumonia has always been a policy priority. As a result, the Government has undertaken several initiatives under the national programme Reproductive, Maternal, Newborn, Child, Adolescent Health and Nutrition (RMNCAH+N), including Integrated Management of Neonatal and Childhood Illnesses (IMNCI); childhood vaccination against Haemophilus influenzae type b (Hib), Pertussis, Pneumococcal conjugate vaccine (PCV), and Measles; promotion of exclusive breastfeeding for 6 months; adequate complementary feeding; reduction in household air pollution; and provision of safe water, sanitation, and hygiene (WASH) [7].

Despite these efforts, inappropriate care-seeking remains a concern in India. A study by Gadapani et al. (2025) reported that while 97% of those with pneumonia symptoms received care from healthcare providers, only 3% received care from government hospitals; 71% received care from non-registered medical practitioners (RMPs) [8]. Addressing this, the government of India launched the 2019 Childhood Pneumonia Management Guidelines, which aim to reduce the healthcare access gap for childhood pneumonia by bridging the community and facility care, thereby improving access to appropriate care [5]. The guidelines recommend active case finding of pneumonia cases by community health workers (Accredited social health activist—ASHA), followed by the appropriate case management at primary health care facilities by trained healthcare workers (Auxiliary nurse midwife—ANM and Community Health officer — CHO) and timely referral of severe cases for management at higher facilities (Community Health Centres — CHCs and District Hospitals — DHs).

If implemented as planned, the new guidelines could potentially bridge the gap in access to appropriate pneumonia care. However, recently conducted implementation research highlighted the key bottlenecks in the implementation of the 2019 guidelines, such as deficient infrastructure and limited space, disrupted medicine supply, lack of specific budget allocation for medicine procurement, absence of functional equipment, staff shortages, lack of training, referral linkage issues, and limited accessibility to guideline materials [9]. The bottlenecks represent challenges at various levels, from policy to implementation, including poor adoption across states and districts, inadequate funding, underutilisation of allocated funds, the existing workload of key personnel involved in programme implementation, and insufficient training.

Despite strong policy intent, there is no empirical evidence on whether the 2019 Childhood Pneumonia Management Guidelines are cost-effective, given India’s limited healthcare resources. In resource-limited settings, robust evidence that encompasses clinical and economic implications will make a strong case for decision-makers to allocate funds to adopt and scale up interventions. Economic evaluations provide a structured, systematic tool for comparing the costs and consequences of alternative interventions and assessing whether additional investment is an efficient use of resources. Cost-effectiveness analysis is a form of economic evaluation that estimates the incremental cost and health gains of one intervention compared with another, summarised as the incremental cost-effectiveness ratio (ICER). The ICER is compared with a context-specific cost-effectiveness threshold to inform resource allocation decisions. Although Cost-effectiveness estimates are available for the WHO Childhood Pneumonia Management Guidelines (2013) [10], no such evidence is available for India’s 2019 Childhood Pneumonia Management Guidelines, which introduce a new service-delivery mechanism to improve equitable access to pneumonia care. This represents a critical evidence gap. Against this backdrop, we evaluated the cost-effectiveness of India’s 2019 Childhood Pneumonia Management Guidelines compared with no treatment from a health system perspective.

## Methodology

### Model description

We parametrised a decision tree model in Microsoft Excel to estimate the incremental cost-effectiveness of India's 2019 Childhood Pneumonia Management Guidelines, assuming universal coverage, compared with no treatment, from a health system perspective in India. No treatment scenario was used as a comparator to estimate the full incremental health gains, particularly to reflect real-world status in rural, resource-constrained settings. The programme targets under-five population and provides treatment strategies for infants aged 0 to 59 days with signs/symptoms of PSBI and children aged 2 to 59 months with signs/symptoms of pneumonia, hence we developed two different model structure to reflect the natural history of disease among infants aged 0 to 59 days with PSBI and child aged 2 to 59 months with pneumonia (see Fig. S1 and S2 in Supplementary File 1).

We estimated the standardised unit cost from a health system perspective using a normative costing approach [11]. Health effects were measured as disability-adjusted life years averted (compared with no treatment), estimated using the WHO DALY calculation template [12]. We adopted the short-term analytic horizon corresponding to the pneumonia episode, while lifetime health losses due to premature deaths were captured through years of life lost (YLL). Importantly, we did not model any long-term morbidity beyond the acute episode. The analysis discounted the future health outcomes and capital costs at 3% to account for time preference. We reported our findings as the incremental cost per DALY averted by implementing the guidelines, compared with no treatment. Incremental analysis was further reported for Indian states and union territories (UT) to inform state-level resource allocation decisions.

### Cost data estimation and analysis

We used a bottom-up micro-costing approach to estimate the health system cost of providing treatment for childhood pneumonia through Health and wellness centres (HWCs), primary health centres (PHCs), community health centres (CHCs), and District hospitals (DHs). We also estimated the costs of providing treatment in these facilities by different providers: Medical Officer (MO), Community Health Officer (CHO), and Auxiliary Nurse-Midwife (ANM). First, we identified relevant cost centres, such as outpatient rooms, inpatient wards, waiting areas, and pharmacies. We quantified the resources/inputs required to treat a child with pneumonia: building space, medicines, personal time, equipment, utilities, inpatient bed service, and transport for referral. Costing followed the normative approach, guided by national guidelines and the Indian Public Health Standards (IPHS) 2022, to define the types and quantities of resources [13].

#### Quantification of resources

Capital resources, such as the outpatient department (OPD) or inpatient ward space, are shared across cases with different illnesses. We apportioned capital resources based on caseloads in accordance with the Minimum performance standards set for key healthcare staff under IPHS 2022 [13]. We assumed healthcare providers spend an average of 10 min per day, and the total personal time per case was estimated by multiplying this per-day time by the treatment duration. Non-consumables required for pneumonia management include an inpatient bed, a pulse oximeter, a stopwatch, a thermometer, a nasal cannula, an oxygen mask, and a nebulisation mask. We included the cost of beds in the treatment cost estimation; however, it was challenging to apportion the cost of other consumables to pneumonia cases. Additionally, these costs would be negligible; hence, excluding them should not bias the cost-effectiveness estimates. For recurrent resources, we multiplied the daily consumption quantity by the treatment duration, then added wastage (20% for medicines; 10% for other consumables) to estimate the amount of resources required per case. Referral costs were included for severe cases referred from primary facilities to CHC or DH. To estimate the cost of ambulance referral, we first determined the quantities of fuel and personal time (nurses and drivers) required to operate an ambulance over 1 km. We assumed an average distance of 10 km between higher-level facilities and primary health care facilities. We estimated utility consumption, such as electricity, water, and internet, as a function of building space consumption [14].

#### Valuing resources

After identifying and measuring resources, we compiled costs from various sources — primarily government, such as government-e-marketplace (for logistics), Pradhan Mantri Jan Aushadhi Price list (for medicines) and job search websites (for human resources) — and estimated unit costs of different resources for the reference year 2024 (see cost analysis methodology in Supplementary File 1 and calculations in Supplementary File 2).

### Health outcome data estimation and analysis

Tables 1 and 2 summarises the epidemiological inputs used in the cost-effectiveness analysis. Published clinical trials, systematic reviews, and meta-analyses informed epidemiological inputs. Due to the lack of recent census data, we used projected state-level population estimates for 2024. As data on the population aged 0–59 days and 2–59 months are not readily available, we made an informed estimate. Assuming a uniform distribution of births throughout the year, infants aged 0 to 59 days would constitute 1/6 of the total population under 1 year. Given that children under 5 years constitute about 9% of the total population [15] and assuming a uniform distribution across age groups, infants (age < 1 year) would account for approximately 1.8% of the total population. Therefore, the proportion of infants aged 0 to 59 days would be approximately 0.3% of the total population. State-wise estimates on the incidence of pneumonia were obtained from the study by Wahl & colleagues (2020) [16].

**Table 1:** Epidemiological parameters: Possible Severe Bacterial Infections (PSBI)

Epidemiological parameters	Value (Lower-upper)	Distribution	Source
Proportion of PSBI cases presenting with fast breathing pneumonia	0.7 (0.56–0.84)	Beta	19–21
Proportion of PSBI cases presenting with clinically severe infection (CSI)	0.2 (0.16–0.24)	Beta	19–21
Proportion of PSBI cases presenting with critical illness (CI)	0.1 (0.08–0.12)	Beta	19–21
**Treatment outcomes**			
**PSBI cases treated with an outpatient regimen**			
**Fast Breathing Pneumonia**			
- Probability of recovery	0.95 (0.87–0.97)	Beta	Pooled estimates (19–21)
- Probability of treatment failure	0.05 (0.03–0.12)	Beta	Pooled estimates (19–21)
- Probability of death among treatment failure cases	0.11 (0.0006-0.71)	Beta	Pooled estimates (19–21)
Clinically Severe Infections (CSI)			
- Probability of recovery	0.94 (0.92–0.96)	Beta	Pooled estimates (19–21)
- Probability of treatment failure	0.06 (0.04–0.08)	Beta	Pooled estimates (19–21)
- Probability of death among treatment failure cases	0.2 (0.11–0.33)	Beta	Pooled estimates (19–21)
Critical Illness (CI)			
- Probability of recovery	0.95 (0.22–0.99)	Beta	Pooled estimates (19–21)
- Probability of treatment failure	0.05 (0.00-0.77)	Beta	Pooled estimates (19–21)
- Probability of death among treatment failure cases	0.64 (0.44–0.79)	Beta	Pooled estimates (19–21)
**PSBI cases treated with an inpatient** ** regimen**			
**Fast Breathing Pneumonia**			
- Probability of recovery	1 (1.0–1.0)	Beta	Pooled estimates (19–21)
- Probability of treatment failure	0 (0.0–0.0)	Beta	Pooled estimates (19–21)
- Probability of death among treatment failure cases	0 (0.0–0.0)	Beta	Pooled estimates (19–21)
Clinically Severe Infections (CSI)			
- Probability of recovery	0.93 (0.87–0.97)	Beta	Pooled estimates (19–21)
- Probability of treatment failure	0.07 (0.03–0.12)	Beta	Pooled estimates (19–21)
- Probability of death among treatment failure cases	0.5 (0.2–0.8)	Beta	Pooled estimates (19–21)
Critical Illness (CI)			
- Probability of recovery	0.74 (0.54–0.87)	Beta	Pooled estimates (19–21)
- Probability of treatment failure	0.25 (0.16–0.46)	Beta	Pooled estimates (19–21)
- Probability of death among treatment failure cases	0.59 (0.38–0.77)	Beta	Pooled estimates (19–21)
No Treatment			
- Probability of dying	0.35 (0.28–0.42)	Beta	20

**Table 2:** Epidemiological parameters: Pneumonia

Epidemiological parameters	Base case	Distribution	Source
**Treatment Group**			
- Probability of treatment failure at day 4	0.075 (0.06–0.09)	Beta	17
- Probability of hypoxia among severe pneumonia/treatment failure cases (Hypoxic Pneumonia)	0.36 (0.35–0.37)	Beta	18
- Probability of dying due to hypoxic pneumonia	0.017 (0.014–0.02)	Beta	18
- Probability of dying due to non-hypoxic pneumonia	0.005 (0.004–0.006)	Beta	18
No Treatment			
- Probability of dying	0.05 (0.03–0.07)	Beta	Assumed

*Treatment failure includes clinical deterioration, relapse and mortality

There were no available effectiveness data for the 2019 Childhood Pneumonia Management Guidelines; however, since the guidelines are based on the 2014 WHO Pneumonia Management Guidelines with a delivery mechanism contextualised to India’s existing healthcare structure, we used the efficacy estimate of the 2014 WHO Guidelines, obtained from WHO’s recently published childhood pneumonia management guidelines [17]. While developing the decision-analytic model for pneumonia, we assumed that a child first develops fast-breathing pneumonia and seeks treatment in accordance with the 2019 guidelines in the Treatment group. Treatment failure in fast-breathing pneumonia would indicate severe pneumonia and thus warrants management according to the guidelines for severe pneumonia in children under five. Data on proportions of children with severe pneumonia developing hypoxia and case fatalities were obtained from the study by Awasthi et al. [18]. There were no evidence on precise estimates of case fatalities in completely untreated cases in India. Evidence suggests that mortality in untreated children with pneumonia can be as high as 20% [19]. However, to avoid overestimating cost-effectiveness, we used a conservative approach and assumed that 5% of cases would die without treatment in the base case.

For PSBI in infants aged 0 to 59 days, we assumed an incidence of 10%, based on estimates from earlier studies [20]. We obtained effectiveness data for inpatient and outpatient treatment regimens for PSBI cases by pooling estimates from three studies conducted in India (Fig. S3 in Supplementary File 1) [20–22]. These were implementation research studies that assessed the effectiveness of inpatient and outpatient regimens across PSBI severity—fast breathing pneumonia (FBP), clinically severe infection (CSI), and critical illness (CI). As no state-specific PSBI incidence estimates were available, we applied the national-level incidence estimate (10%) to all states and performed state-wise incremental analysis.

To estimate DALYs, we used the median age of death due to pneumonia in children under five as 8.9 months, and for PSBI, we assumed the median age of death as 26 days [23]. Disability weight was assigned a value of 0.21 — the standard for infectious disease — whereas for those who died of pneumonia, it was assigned a value of 1 [10]. We estimated the incremental cost and effectiveness of treating pneumonia across healthcare facility levels by subtracting the cost and effectiveness of the treatment arm from those of the no-treatment arm, respectively, and then dividing the incremental cost by the incremental effectiveness to estimate the ICER.

$$\:ICER=\frac{Cost\left(Treatment\right)-cost\:\left(No\:Treatment\right)}{(benefit\left(Treatment\right)-benefit(No\:Treatment)}$$

### Sensitivity analysis

To estimate the joint effect of parameter uncertainty on the cost-effectiveness estimates, we conducted a probabilistic sensitivity analysis using 10,000 Monte Carlo simulations. We used 95% confidence intervals for parameters reported in the published studies to define the lower and upper limits; otherwise, we used ± 20% variation around base-case estimates. We presented our PSA findings using the cost-effectiveness plane and the cost-effectiveness acceptability curve (CEAC). We performed all analyses in R. 

### Ethics declaration

Not applicable.

## Results

Table S1 (Supplementary file 1) summarises the costs of treating pneumonia, severe pneumonia, and PSBI across different levels of health care facilities in India. Based on the scenarios described in Table S2 (Supplementary file 1), we estimated cost parameters for cost-effectiveness analysis [Table 3]. Based on our analysis, the estimated cost of treating pneumonia without treatment failure (i.e., only fast-breathing pneumonia) is USD 3, whereas the treatment cost increases to USD 107 in cases of treatment failure. The presence of hypoxia in such children further raises the treatment cost to USD 113.

**Table 3:** Cost parameters

Cost parameters	Base case (USD)	Distribution
**Pneumonia**		
- Without Treatment Failure (Fast Breathing Pneumonia)	3 (2.94–3.6)	Log-normal
- With Treatment Failure (Severe Pneumonia)		
Hypoxia	113 (91–135)	Log-normal
No hypoxia	107 (86–128)	Log-normal
Possible Serious Bacterial Infection (PSBI) - Without Treatment failure		
Outpatient regimen	14 (11.2–16.8)	Log-normal
Inpatient regimen	105 (84–126)	Log-normal
- With Treatment failure		
Outpatient regimen	132 (106–158)	Log-normal
Inpatient regimen	164 (161–241)	Log-normal

The estimated cost of treating PSBI with an outpatient regimen is USD 14, which increases to USD 132 when a child suffers treatment failure. Treating PSBI with an inpatient regimen cost USD 105, which increases to USD 201 in case of treatment failure.

Table 4 presents the incremental analysis. Treating pneumonia in accordance with the guidelines incurs an incremental cost of USD 7.8 per DALY averted, which is equivalent to 0.3% of India’s 2024 per capita Gross Domestic Product (GDP), i.e., USD 2695 [24].

**Table 4:** Incremental analysis

	Incremental cost (USD)	Incremental benefit (DALYs averted)	ICER (USD)	% of per-capita GDP 2024
Possible Serious Bacterial Infection (PSBI) in infants aged 0 to 59 days				
- Fast breathing pneumonia				
Outpatient regimen	20	9.8	2.02	0.07%
Inpatient regimen	105	10	10.5	0.38%
- Clinically severe infections				
Outpatient regimen	21	9.7	2.18	0.08%
Inpatient regimen	112	9	12.4	0.46%
- Critical illness				
Outpatient regimen	20	9.1	2.19	0.08%
Inpatient regimen	128	5.8	22.4	0.83%
- PSBI (Overall)				
Outpatient regimen	20	9.7	2.07	0.07%
Inpatient regimen	108	9.4	11.6	0.43%
Pneumonia in children aged 2 to 59 months	10.9	1.42	7.83	0.29%

For PSBI, the cost-effectiveness varies by severity and treatment regimen. When managed through the outpatient regimen, the additional cost per DALY averted (ICER) is USD 2.1 (0.07% of per-capita GDP; 2024), while the inpatient regimen costs USD 11.6 per DALY averted (0.43% of per-capita GDP; 2024). The ICER varies with PSBI severity, reflecting differences in the probabilities of treatment success and failure across severity levels and treatment strategies. Tables S3 and S4 (Supplementary file 1) present incremental analysis for states/UTs in India.

**Fig. 1:**
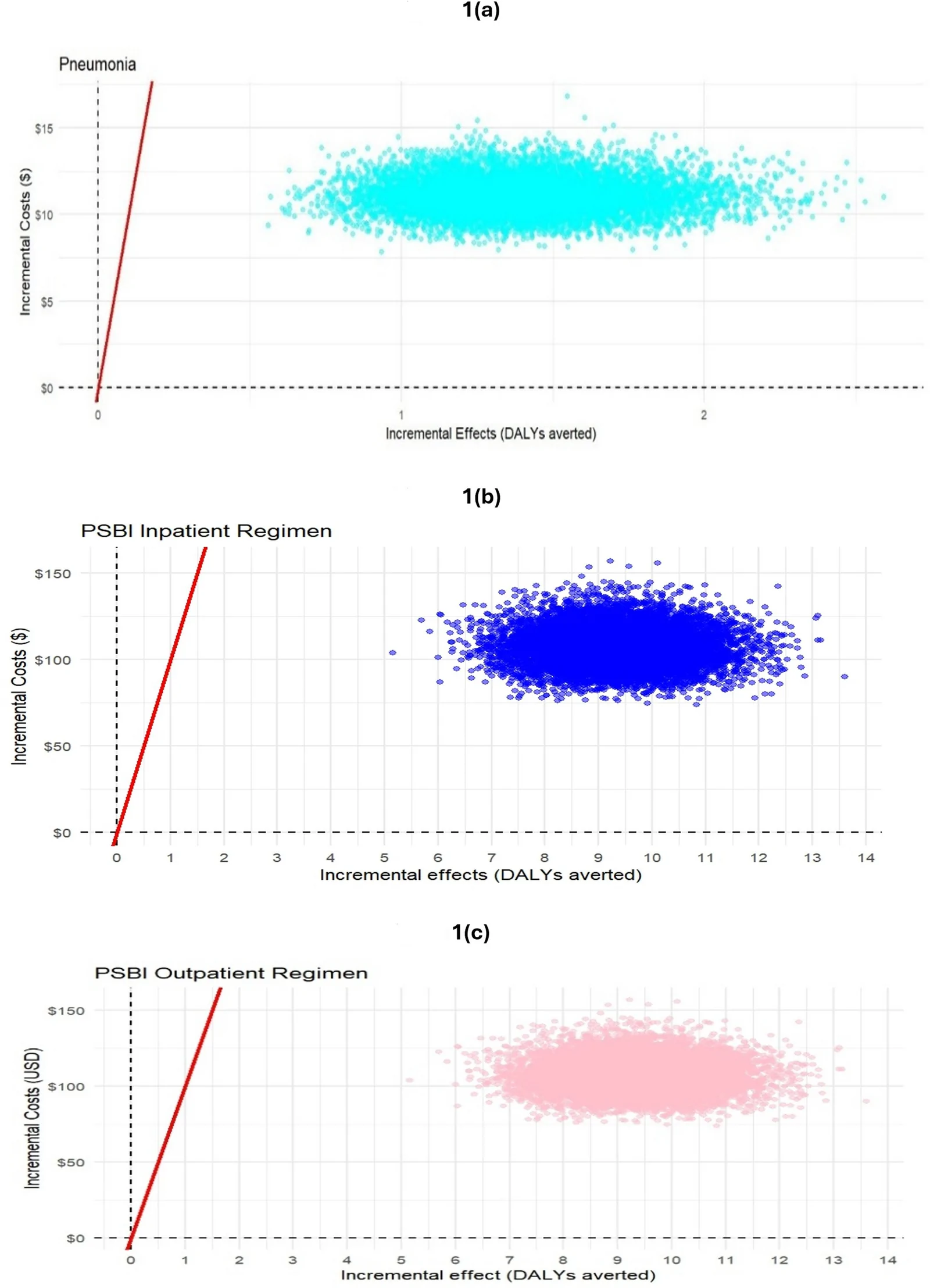
Figure 1(**a**-**c**). Cost-effectiveness planes showing results of probabilistic sensitivity analysis (PSA) for India’s 2019 Childhood Pneumonia Management Guidelines (Pneumonia and Possible Serious Bacterial Infection (PSBI)) compared with no treatment. The x-axis displays incremental effectiveness, measured as disability-adjusted life years (DALYs) averted for the treatment group relative to no treatment. The y-axis indicates incremental cost in USD. Each dot corresponds to a single simulation in which input parameters were varied probabilistically to estimate overall result uncertainty. The diagonal red line represents the willingness-to-pay (WTP) threshold at USD 100, which is substantially lower than the recommended WTP for India (approximately USD 2695, equivalent to per-capita GDP in 2024). Each dot illustrates the incremental cost-effectiveness ratio (ICER), calculated as incremental costs divided by incremental effectiveness. Results were considered cost-effective if the ICER, represented by a dot in the North-East quadrant, fell below the WTP threshold while still incurring some costs

**Fig. 2:**
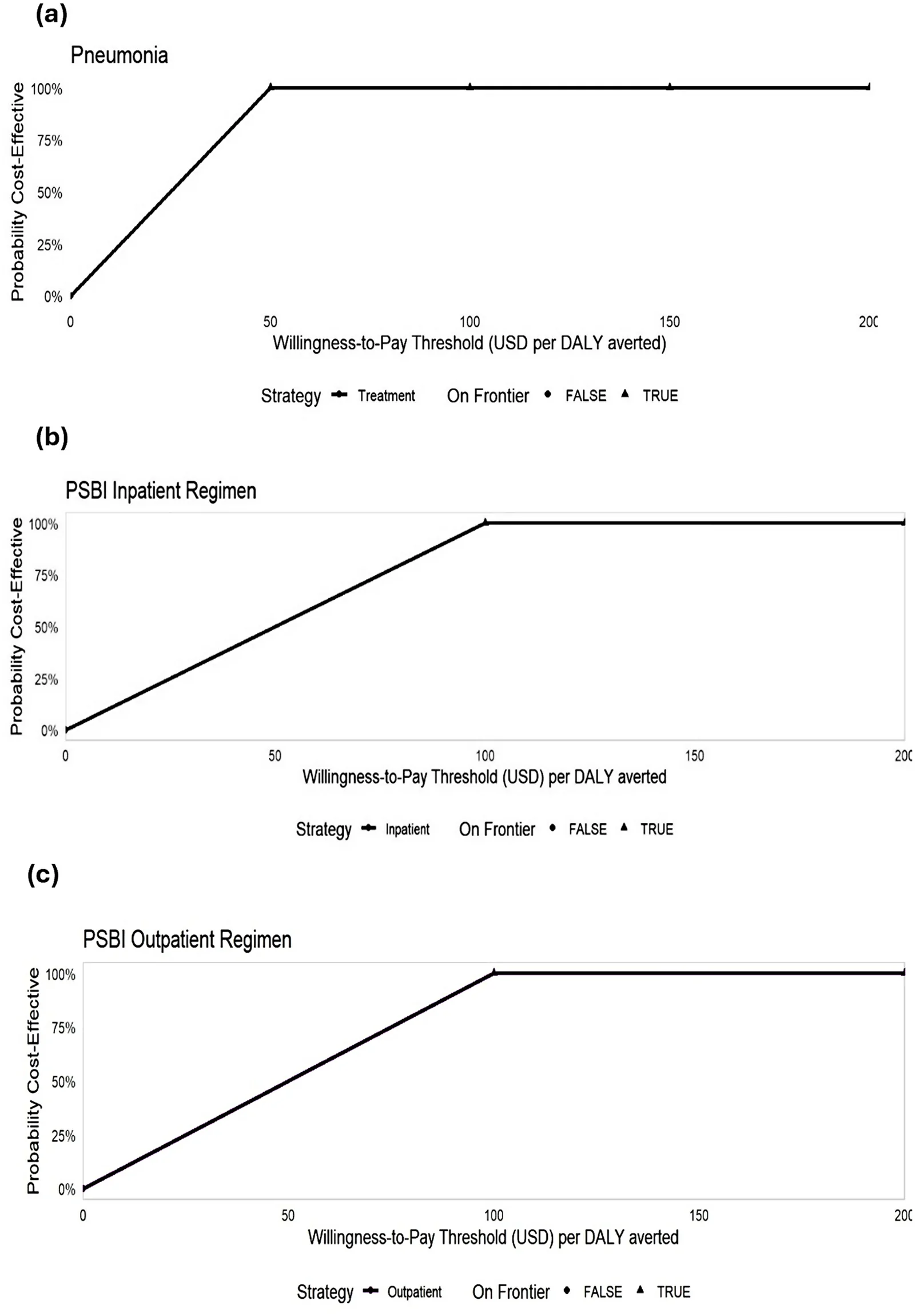
(**a**-**c**). The cost-effectiveness acceptability curve (CEAC) shows the results of the probabilistic sensitivity analysis (PSA) for India’s 2019 Childhood Pneumonia Management Guidelines, compared with no treatment. The y-axis shows the probability that the intervention remains cost-effective at a given willingness-to-pay (WTP) threshold, while the x-axis shows Willingness-to-Pay (WTP) in USD. The CEAC summarises the number of simulations in which the incremental cost-effectiveness ratio (ICER), as shown by each dot in the cost-effectiveness (CE) plane in Fig. 1, falls below the WTP threshold, thereby indicating cost-effectiveness

Probabilistic sensitivity analysis indicates a 100% probability that the guidelines are cost-effective compared with no treatment, with willingness-to-pay (WTP) thresholds as low as USD 50 for pneumonia management and USD 100 for PSBI, both for inpatient and outpatient management (Figs. 1 and 2)*.*

## Discussion

The present study demonstrates that India’s 2019 Childhood Pneumonia Management Guidelines are cost-effective relative to no treatment. With an incremental cost of treating pneumonia at USD 10.9 and an incremental benefit of 1.42 DALYs averted, the Government of India would incur an additional cost of USD 7.83 to prevent one DALY by implementing the 2019 guidelines, compared to no treatment. Treating PSBI cases in an outpatient setting would cost the government an additional USD 2.1 per DALY averted, whereas treating these cases in an inpatient setting would cost an additional USD 11.6 per DALY averted.

These additional costs per DALY averted represent < 1% of per-capita GDP, indicating that the 2019 guidelines are highly cost-effective.

The Health Technology Assessment in India (HTAIn) — an HTA institution established under the Department of Health Research (DHR), Ministry of Health and Family Welfare (MoHFW), entrusted with synthesising evidence on the clinical and cost-effectiveness of health technologies — recommends using per-capita GDP as a cost-effectiveness threshold [25, 26]. This threshold is used in various published studies conducted under the HTAIn [27, 28]. However, it is important to highlight that using per-capita GDP as the threshold for assessing an intervention as cost-effective is increasingly being questioned in the health economics literature. It consistently argues that the threshold should be much lower than per capita GDP. A study by Pichon-Riviere & colleagues (2023) [29] proposed a simplified approach of estimating country-specific cost-effectiveness threshold based on per-capita health expenditures and life expectancy at birth (or healthy life expectancy), and applied the framework to 174 countries, including India. The study estimated the central value of the threshold per QALY gained to be 0.24 times per capita GDP (95% Confidence interval: 0.12–0.30). The ICER estimates in our study remain substantially below these more conservative threshold estimates, suggesting that the conclusion regarding the cost-effectiveness of the 2019 guidelines is robust to alternative threshold assumptions.

The findings may support the current discourse on optimising the place of treatment and on switching recommendations from hospital-based (standard care) to outpatient-based management; however, the decision will be contingent on treatment outcomes. This may increase access to PSBI management, particularly in low-resource, high-burden settings, where it is currently below 20% [30].

The study also provides aggregated state- and national-level costs and health benefits associated with implementing the 2019 guidelines, using available estimates of pneumonia burden reported by Wahl and colleagues in 2015 [16]. These estimates can guide state-level resource allocation, thereby increasing the policy relevance of the findings for decentralised health planning.

Since this is the first study to analyse the cost-effectiveness of the India’s 2019 Childhood Pneumonia Management Guidelines, direct comparisons with previously published studies are limited. Zhang & colleagues [10] conducted a cost-effectiveness analysis comparing the 2013 WHO childhood pneumonia management guidelines with no treatment across 74 countries, including India, concluding the strategy as cost-effective at an ICER of USD 65 per DALY averted. The Zhang study included both Human Immunodeficiency Virus (HIV) + and HIV -, while the present analysis modelled the estimates for pneumonia in HIV- under-five children. However, given the very low HIV prevalence in children, the cost attributed to HIV + cases was negligible (3% of the total cost).

In this study, costs were estimated normatively, identifying and measuring resources using the IPHS 2022 and 2019 Childhood Pneumonia Management Guidelines. Although a direct comparison is challenging, our estimates are reasonably comparable to those reported in the “Cost of Health Services in India” (CHSI) 2022 study [31]. The CHSI 2022 study estimated costs using a national sample of 38 public and 16 private hospitals across different levels (secondary and tertiary, inpatient and outpatient) of the Indian health system. The study included 327 specialities across tertiary, private, and district hospitals and estimated unit prices per service output at cost-centre levels—outpatient, inpatient, operating theatre, and intensive care unit. After adjusting for inflation, 7-day inpatient bed-days would cost $100 (irrespective of service type) at a district-level hospital, which is comparable to the present study’s estimates that treating a child with pneumonia with a 7-day inpatient admission would cost between USD 111 (no hypoxia) and 117 (with hypoxia).

In the present results, treating fast-breathing pneumonia costs USD 3 at the District level, assuming 2 days of OPD visits; however, according to CHSI 2022, the inflation-adjusted average OPD cost for a 2-day treatment (irrespective of the service type) at the District Hospital level is USD 7.3. The fact that the CHSI 2022 study estimated costs across a heterogeneous group of disease conditions and populations accounts for the differences between CHSI’s cost estimates and those reported in our study.

Methodologically, the use of a decision-analytic model enabled the systematic synthesis of relevant evidence from multiple sources and facilitated comparison between treatment and non-treatment scenarios. To address parameter uncertainty, the study employed probabilistic sensitivity analysis, which confirmed the robustness of the findings. The probability of the intervention being cost-effective was 100% at a willingness-to-pay of USD 100 per DALY averted for PSBI and USD 50 per DALY averted for pneumonia. These values are well below the cost-effectiveness threshold, i.e., per capita GDP in India and the threshold proposed by Pichon-Riviere & colleagues (2023). The study also provides incremental costs and benefits for Indian states and UTs to inform state-level resource allocation, which increases the policy relevance of the findings for decentralised health planning.

The study has certain limitations. We estimated the cost-effectiveness of the 2019 Childhood Pneumonia Management Guidelines compared with no treatment, which, however, does not represent standard care in India but still reflects the true scenario in most rural and remote geographies. Therefore, the findings provide policy-relevant evidence on potential health gains and additional costs of expanding pneumonia care services in these settings, which would help reduce existing inequalities in access.

Since efficacy or effectiveness data for the 2019 Childhood Pneumonia Management Guidelines are unavailable, we used efficacy estimates from studies comparing antibiotics recommended under the guidelines with no antibiotic treatment. Using efficacy rather than effectiveness to estimate health benefits, choosing no treatment as the comparator and assuming 100% coverage may have led to an overestimation of the incremental health benefits.

Another major limitation is that the study employed a normative costing framework and estimated the resources required to deliver the pneumonia care in accordance with guideline recommendations. However, implementation research [9] has highlighted important barriers in the delivery of childhood pneumonia care, including an inadequate workforce, interrupted supply of medicines and other consumables, referral challenges and infrastructure constraints. Similar challenges have been identified in the Disease Control Priorities (DCP) literature [32], which emphasises that the effectiveness of child health programmes depends on broader health-system functions, including the trained workforce, supervision and monitoring, supply and logistics management, and infrastructure. To achieve the level of service delivery assumed in the model, system-level strengthening would require additional investment in workforce capacity, training, supervision, programme management, supply chain management, and infrastructure that were not explicitly included in the analysis. Consequently, the programme cost under routine implementation may exceed the estimate in the analysis. This might have resulted in a modest overestimation of the intervention’s cost-effectiveness. However, the current ICER is substantially below the recommended thresholds; inclusion of these costs is unlikely to alter the findings.

Moreover, these investments in system-level strengthening would not only benefit the childhood pneumonia programme but also improve the delivery of other child health interventions. Therefore, while the inclusion of health system strengthening costs may increase the overall programme cost, the associated health benefits would extend beyond the pneumonia-specific outcome estimated in the present model.

Accordingly, the reported ICER should be interpreted as the expected cost-effectiveness under the assumptions incorporated into the model, rather than the cost-effectiveness that may be observed during routine implementation. The extent to which these projected health benefits estimated in the present analysis are realised in practice will depend on treatment coverage. adherence to guideline recommendations, medicine availability, referral completion, and the broader functioning of the health system.

Another limitation is the lack of recent state-level estimates of Pneumonia incidence. The current state-level estimates are based on 2015 data, and changes in pneumonia epidemiology over time may affect the absolute number of cases and thus influence the projected aggregated state- and national-level costs and health benefits, though they will not change the patient-level incremental analysis. To address this, we also estimated aggregated costs and benefits using the latest national-level estimates of Pneumonia incidence, although the unavailability of such estimates for states limited the state-level analysis.

In conclusion, based on the current analysis, the 2019 Childhood Pneumonia Management Guidelines present a cost-effective and scalable strategy and warrant adoption and scale-up nationwide and across states, particularly in resource-constrained settings. However, the study should be interpreted in light of the assumptions incorporated into the model, including treatment coverage, effectiveness estimates, choice of comparators, and implementation costs. Despite these limitations, the results provide important economic evidence to inform policy decisions to improve access to pneumonia care and thus accelerate progress towards overall child health goals.

## Supplementary Information

Below is the link to the electronic supplementary material.

Supplementary Material 1

Supplementary Material 2

## Data Availability

The dataset generated and/or analysed during the current study has been uploaded as supplementary material.
