# Supplementary material for: Bridging community and facility care: cost-effectiveness of implementing 2019 childhood pneumonia management guidelines in India
Source: Cost Eff Resour Alloc. 2026 Jul 2;24:116. doi: 10.1186/s12962-026-00792-3 (PMC13599094; doi:10.1186/s12962-026-00792-3)
Supplement: Supplementary file 1 [file 12962_2026_792_MOESM1_ESM.docx]

Contents

[Figure S1. Decision tree model for pneumonia management in children aged 2 to 59 months in India	2](#_Toc233619480)

[Figure S2. Decision tree model for Possible serious bacterial infection (PSBI) management in children aged 0 to 59 days in India	3](#_Toc233619481)

[Cost analysis methodology	4](#_Toc233619482)

[Figure S3 (a). Pooled estimates of the probability of recovery among children with PSBI on treatment	8](#_Toc233619483)

[Figure S3 (b). Pooled estimates of the probability of treatment failure among children with PSBI on treatment	9](#_Toc233619484)

[Figure S3 (c). Pooled estimates of the probability of dying among treatment failure PSBI cases	10](#_Toc233619485)

[Table S1. Cost of treating pneumonia/PSBI in children under five from India’s health system perspective, as per the 2019 Childhood Pneumonia Management Guidelines	11](#_Toc233619486)

[Table S2. Treatment Pathways based on Childhood Pneumonia Management Guidelines 2019	12](#_Toc233619487)

[Table S3. State-wise estimates of incremental cost and incremental benefits of treating possible serious bacterial infections (PSBI) in children aged 2 to 59 days, as per India’s 2019 Childhood Pneumonia Management Guidelines, compared with no treatment.	13](#_Toc233619488)

[Table S4. State-wise estimates of incremental cost and incremental benefits of treating pneumonia in children aged 2 to 59 months, as per India’s 2019 Childhood Pneumonia Management Guidelines, compared with no treatment.	14](#_Toc233619489)

[References	14](#_Toc233619490)

# Figure S1. Decision tree model for pneumonia management in children aged 2 to 59 months in India

Child aged 2-59 months presenting with pneumonia

Treatment

Recovered

Treatment failure/hospitaliation

Hypoxia

Recovered

Died

No hypoxia

Recovered

Died

No treatment

Recovered

Died

# Figure S2. Decision tree model for Possible serious bacterial infection (PSBI) management in children aged 0 to 59 days in India

PSBI case

Treatment

Fast breathing penumonia

Outpatient treatment at HWC

Recovered

Treatment failure

Recovered

Died

Inpatient treatment at CHC/DH

Recovered

Treatment failure

Recovered

Died

Clinical severe infections

Outpatient treatment at HWC

Recovered

Treatment failure

Recovered

Died

Inpatient treatment at CHC/DH

Recovered

Treatment failure

Recovered

Died

Critical illness

Outpatient treatment at HWC

Recovered

Treatment Failure

Recovered

Died

Inpatient treatment at CHC/DH

Recovered

Treatment Failure

Recovered

Died

No treatment

Recovered

Died

# Cost analysis methodology

**Estimation of building space cost per case**

*Suppose*

- i: index for each cost centre (e.g., OPD, pharmacy, etc.)
- Si: building space (sq. ft) for cost centre i
- R: rental price per sq. ft (₹/sq. ft/day)
- Pi: patient load per day at cost centre i (based on IPHS 2022 standards)
- D: number of days of treatment per case

i. Identified cost centres (i) e.g., OPD, waiting area, pharmacy, inpatient ward.
ii. Estimated building space (Si) (in square feet) for each cost centre using IPHS infrastructure layout 2022.
iii. Multiplied building space for each cost centre separately by the rental price per square foot to calculate the rental price for each cost centre.

*Ci=Si ×R*

where Ci is the daily rental cost for cost centre i.

iv. Apportioned rental price for each cost centre by patient load per day as per Minimum performance standard IPHS 2022, to calculate the cost of building space per case per day for each cost centre.

$$ci=Ci/Pi$$

Where Pi is the patient load per day.

v. Added per-case per-day rental price for each cost centre to estimate building space per case per day.

$$ctotal=\sum_{i} ci$$

vi. Multiplied building space per case per day by days of treatment

$$Building space cost per case=ctotal*D$$

Equation 1

**Estimation of Bed cost per case:**

The cost of purchasing a bed and bedding was annualised using the discount factor and shelf life, then divided by 365 days to estimate the per-day bed cost. We multiplied the per-day bed cost by the duration of inpatient treatment to determine the per-case bed cost.

C = Cost of bed and beddings

r = Discount factor

n = Shelf life of bed and beddings

D = duration of treatment

i. Estimated annualised factor using r and n

$$Annualization factor=(r*(1+r)^n)/((1+r)^n-1)$$

ii. Multiplied the cost of beds and bedding by the annualization factor

$$Annualized cost of bed and beddings=C*annualization factor$$

iii. Estimated per-day bed cost

$$Per day bed cost=((Annualized cost))/365$$

iv. Estimated per-case bed cost

$$Per case bed cost=D*Per day bed cost$$

**Estimation of personal cost per case:**

To estimate personal cost per case, we ascertained the salary per minute for each healthcare provider by dividing their monthly salary by working days in a month, working hours in a day and total minutes in an hour (assuming twenty-four working days and 6 hours a day); we further determined the personal cost per day for each provider by multiplying salary per minute with total personal time per day and summed up personal cost for each provider to estimate personal cost per case per day. Finally, we multiplied the per-case per-day cost by the number of treatment days to obtain the per-case personal cost.

Suppose

W = number of working days per month

H=number of working hours per day

M = number of minutes per hour

Si = Salary per month for healthcare worker i

Ti = personal time per case by healthcare worker i

i. = Salary per minute for healthcare worker i

$$si=Si/(W*H*M)$$

ii. Personal cost per case per day for healthcare worker i (*PCi*)

$$PCi =si* Ti$$

iii. Personal cost per case per day (*PCtotal*)

$$PCtotal= \sum_{i} PCi$$

iv. Personal cost per case

$$Persoanl cost per case=D*\sum_{i} PCi$$

Equation 2.

**Estimation of recurrent cost per case**

Recurrent costs such as drugs and consumables were calculated as follows: (i) multiplying the unit cost of drugs/consumables by the quantity required per day; (ii) followed by summing up cost of drugs/consumables to estimate drugs/consumables cost per case per day; (iii) finally multiplying this by days of treatment to estimate drugs/consumable cost (recurrent cost) per case.

Pi = cost of drug or consumable i

Qi=quantity of drug/consumable i required per day (wastage added)

D = Duration of treatment

$$Recurrent cost per case=D*\sum_{i} (Pi*Qi)$$

Equation 3.

**Estimation of referral costs**

We estimated referral costs from the health system perspective by calculating the per-km operational cost of running an ambulance, based on fuel and personal time (driver and nurse), and multiplying it by the number of kilometres (distance between home and the healthcare facility).

C = Cost per litre (year 2023)

A = Average mileage, i.e., KM per litre

T = Time taken to cover 1 KM (same for each person accompanying the case during transport)

i. Fuel cost per km

$$fuel cost per KM (F)=C/A$$

ii. Personal cost per KM (used the same equation used in personal cost estimation)

$$Personal cost per KM (Pi)= si*T$$

*iii. Operational cost of ambulance per KM*

$$Operational cost of ambulance per KM =F+ \sum_{i} Pi$$

iv. We assumed a 10 Km average distance to estimate referral cost.

$$Cost of ambulence=10*Operational cost of ambulence per KM$$

Equation 4.

**Estimation of utility costs per case**

We considered an average of 4% of the total building space cost per case as the utility cost.

$$Utility cost per case=0.04*Building space cost per case$$

Equation 5

**Estimation of Treatment cost per case**

Finally, we summed the costs of each required resource per case to estimate the treatment cost per case of pneumonia/severe pneumonia/PSBI.

$$cost of treatment per case= Building space cost per case+Personal cost per case+Recurrent cost per case+Bed-cost per case (for inpatient treatment)+utility cost+Referral cost (for severe cases)$$

*Equation 6*

# Figure S3 (a). Pooled estimates of the probability of recovery among children with PSBI on treatment

Figure S3 (b). Pooled estimates of the probability of treatment failure among children with PSBI on treatment

Figure S3 (c). Pooled estimates of the probability of dying among treatment failure PSBI cases

# Table S1. Cost of treating pneumonia/PSBI in children under five from India’s health system perspective, as per the 2019 Childhood Pneumonia Management Guidelines

| Disease | Level of care | Intervention | Personal | Unit cost  (USD) |
| --- | --- | --- | --- | --- |
| PSBI in infants aged 0 to 59 days | HWC | 7 days of treatment with oral amoxicillin and injection (inj.) Gentamycin | ANM/CHO | 14 |
|  | CHC | 7 days of treatment with injection. ampicillin and inj. Gentamycin | Medical officer/ Paediatrician and nurses | 99 |
|  | DH | 7 days of treatment with injection. ampicillin and inj. Gentamycin | Medical officer/Paediatrician and nurses | 111 |
| Treatment Failure PSBI cases (initially treated with an outpatient regimen) | CHC/DH | 3 days treatment with outpatient regimen + 7 days treatment with Inpatient regimen | Medical officer/Paediatrician and nurses | 132 |
|  |  |  |  |  |
| Treatment Failure PSBI cases (initially treated with inpatient regimen) | CHC/DH | Three days of inpatient care with standard inpatient regimen, followed by 7 days of treatment with IV Ceftriaxone | Medical officer/Paediatrician and nurses | 165 |
| Fast Breathing Pneumonia | HWC | 5 days of treatment with oral amoxicillin | ANM/CHO | 3 |
|  | CHC | 5 days of treatment with oral amoxicillin | Medical officers/paediatricians, and nurses | 3 |
|  | DH | 5 days of treatment with oral amoxicillin | Medical officers/paediatricians, and nurses | 3 |
| Severe Pneumonia | CHC  (without Hypoxia) | 7 days of treatment with injection. ampicillin and inj. Gentamycin | Medical officers/paediatricians, and nurses | 97 |
|  | CHC (with Hypoxia) | 7 days of treatment with injection. ampicillin and inj. Gentamycin | Medical officers/paediatricians, and nurses | 103 |
|  | DH  (without Hypoxia) | 7 days of treatment with injection. ampicillin and inj. Gentamycin | Medical officers/paediatricians, and nurses | 111 |
|  | DH (with Hypoxia) | 7 days of treatment with injection. ampicillin and inj. Gentamycin | Medical officers/paediatricians, and nurses | 117 |

HWC = Health and Wellness Centre; CHC= Community Health Centres; DH= District Hospitals

# Table S2. Treatment Pathways based on Childhood Pneumonia Management Guidelines 2019

| Scenarios | Scenario | Assumptions |  |
| --- | --- | --- | --- |
| Pneumonia | Treating pneumonia at HWC by ANM/CHO and referring treatment failure/severe cases to CHC/DH | - All cases to receive an outpatient regimen at the Health and Wellness Centre. - At HWC, the caseload is to be equally shared between ANM and CHO (1:1) - We assumed all treatment failures would be a severe form of pneumonia and, therefore, would follow the line of management recommended for severe pneumonia under the guidelines. - All treatment failures/severe cases would be referred to the hospital and incur the additional cost due to hospital care. - All cases to be distributed equally (1:1) between CHC and DH. At each facility, the caseload is to be equally shared between the medical officer and the Paediatrician - Estimated cost of treating treatment failure/severe cases at hospital would be an average of the cost of treating pneumonia at CHC and DH; Cost is estimated separately for cases with hypoxia and without hypoxia [ Refer Equation 7]. | - 5 days of treatment with oral amoxicillin for pneumonia, with only fast breathing - In case of treatment failure/severe pneumonia, refer to CHC/DH for inpatient management. At CHC/DH, treatment includes 7 days of treatment with injection. ampicillin and inj. Gentamycin |
| Possible serious bacterial infection | **When referral is not feasible**  **Outpatient regimen:** Treatment at HWC by ANM/CHO using an outpatient treatment regimen  Treatment failure cases to be referred to CHC/DH | - All cases will receive an outpatient regimen at the Health and Wellness Centre. At HWC, the caseload is to be equally shared between ANM and CHO (1:1) - We assumed all treatment failure cases would be referred to the hospital and incur the additional cost due to hospital care. - All cases to be distributed equally (1:1) between CHC and DH, - At each facility, the caseload is to be equally shared between the medical officer and the Paediatrician - Estimated cost of treating PSBI at the hospital would be an average of the cost of treating PSBI at CHC and DH [Refer Equation 7] | - 7 days treatment with inj. Ampicillin and inj. Gentamycin - In case of treatment failure, the antibiotics of choice: Third level of cephalosporin |
| Possible serious bacterial infection | **Inpatient regimen** Treatment at CHC/DH by a Medical officer/Paediatrician using an Inpatient treatment regime | - All cases to be treated in hospital - Caseload to be equally shared by CHC and DH (1:1) - At each facility, the caseload is to be equally shared between the medical officer and the Paediatrician - Estimated cost of treating PSBI at the hospital would be an average of the cost of treating PSBI at CHC and DH [Refer Equation 7] | 7 days of treatment with inj. Ampicillin and inj. Gentamycin |

*Equation 7. Cost of treatment with an outpatient regimen = cost of treating at HWC + probability of treatment failure* cost of treating failure cases at CHC/DH with an inpatient regimen

# Table S3. State-wise estimates of incremental cost and incremental benefits of treating possible serious bacterial infections (PSBI) in children aged 2 to 59 days, as per India’s 2019 Childhood Pneumonia Management Guidelines, compared with no treatment.

| **States** | **Total Population** | **Population 2 to 59 days** | **Annual Incidence of PSBI cases [10% Risk]** | **PSBI**  **(Inpatient regimen)** | | **PSBI**  **(Outpatient regimen)** | |
| --- | --- | --- | --- | --- | --- | --- | --- |
|  |  |  |  | **Incremental cost (USD)** | **Incremental benefit (DALYs averted)** | **Incremental cost (USD)** | **Incremental benefit (DALYs averted)** |
| Andhra Pradesh and Telangana | 53,402,000 | 160,206 | 16,021 | 1,730,225 | 150,594 | 320,412 | 155,400 |
| Assam | 36,159,000 | 108477 | 10848 | 1171552 | 101968 | 216954 | 105223 |
| Bihar | 129,205,000 | 387615 | 38762 | 4186242 | 364358 | 775230 | 375987 |
| Chhattisgarh | 30,638,000 | 91914 | 9191 | 992671 | 86399 | 183828 | 89157 |
| Delhi | 21,884,000 | 65652 | 6565 | 709042 | 61713 | 131304 | 63682 |
| Goa | 1,585,000 | 4755 | 476 | 51354 | 4470 | 9510 | 4612 |
| Gujarat | 72,653,000 | 217959 | 21796 | 2353957 | 204881 | 435918 | 211420 |
| Haryana | 30,694,000 | 92082 | 9208 | 994486 | 86557 | 184164 | 89320 |
| Himachal Pradesh | 7,518,000 | 22554 | 2255 | 243583 | 21201 | 45108 | 21877 |
| Jammu and Kashmir | 13,733,000 | 41199 | 4120 | 444949 | 38727 | 82398 | 39963 |
| Jharkhand | 40,129,000 | 120387 | 12039 | 1300180 | 113164 | 240774 | 116775 |
| Karnataka | 68,256,000 | 204768 | 20477 | 2211494 | 192482 | 409536 | 198625 |
| Kerala | 35,967,000 | 107901 | 10790 | 1165331 | 101427 | 215802 | 104664 |
| Madhya Pradesh | 87,954,000 | 263862 | 26386 | 2849710 | 248030 | 527724 | 255946 |
| Maharashtra | 87,954,000 | 263862 | 26386 | 2849710 | 248030 | 527724 | 255946 |
| Odisha | 46,663,000 | 139989 | 13999 | 1511881 | 131590 | 279978 | 135789 |
| Punjab | 30,992,000 | 92976 | 9298 | 1004141 | 87397 | 185952 | 90187 |
| Rajasthan | 82,188,000 | 246564 | 24656 | 2662891 | 231770 | 493128 | 239167 |
| Tamil Nadu | 77,165,000 | 231495 | 23150 | 2500146 | 217605 | 462990 | 224550 |
| Uttar Pradesh | 238,875,000 | 716625 | 71663 | 7739550 | 673628 | 1433250 | 695126 |
| Uttarakhand | 11,795,000 | 35385 | 3539 | 382158 | 33262 | 70770 | 34323 |
| West Bengal | 99,723,000 | 299169 | 29917 | 3231025 | 281219 | 598338 | 290194 |
| India | 1430000000 | 4290000 | 429000 | 46332000 | 4032600 | 8580000 | 4161300 |

# Table S4. State-wise estimates of incremental cost and incremental benefits of treating pneumonia in children aged 2 to 59 months, as per India’s 2019 Childhood Pneumonia Management Guidelines, compared with no treatment.

| **States** | **Total Population** | **Population 2 to 59 months** | **Incidence rate (per child year)**  [1] | **Annual Incidence of Pneumonia** | **Pneumonia** | |
| --- | --- | --- | --- | --- | --- | --- |
|  |  |  |  |  | **Incremental cost (USD)** | **Incremental benefit (DALYs averted)** |
| Andhra Pradesh and Telangana | 53,402,000 | 4645974 | 0.275 | 1277643 | 13926307 | 3602953 |
| Assam | 36,159,000 | 3145833 | 0.350 | 1101042 | 12001353 | 3104937 |
| Bihar | 129,205,000 | 11240835 | 0.498 | 5597936 | 61017501 | 15786179 |
| Chhattisgarh | 30,638,000 | 2665506 | 0.401 | 1068868 | 11650660 | 3014207 |
| Delhi | 21,884,000 | 1903908 | 0.285 | 542614 | 5914490 | 1530171 |
| Goa | 1,585,000 | 137895 | 0.203 | 27993 | 305120 | 78939 |
| Gujarat | 72,653,000 | 6320811 | 0.493 | 3116160 | 33966142 | 8787571 |
| Haryana | 30,694,000 | 2670378 | 0.326 | 870543 | 9488921 | 2454932 |
| Himachal Pradesh | 7,518,000 | 654066 | 0.320 | 209301 | 2281382 | 590229 |
| Jammu and Kashmir | 13,733,000 | 1194771 | 0.218 | 260460 | 2839015 | 734497 |
| Jharkhand | 40,129,000 | 3491223 | 0.464 | 1619927 | 17657209 | 4568195 |
| Karnataka | 68,256,000 | 5938272 | 0.362 | 2149654 | 23431234 | 6062026 |
| Kerala | 35,967,000 | 3129129 | 0.137 | 428691 | 4672728 | 1208908 |
| Madhya Pradesh | 87,954,000 | 7651998 | 0.563 | 4308075 | 46958016 | 12148771 |
| Maharashtra | 87,954,000 | 7651998 | 0.431 | 3298011 | 35948321 | 9300391 |
| Odisha | 46,663,000 | 4059681 | 0.394 | 1599514 | 17434706 | 4510630 |
| Punjab | 30,992,000 | 2696304 | 0.258 | 695646 | 7582546 | 1961723 |
| Rajasthan | 82,188,000 | 7150356 | 0.499 | 3568028 | 38891501 | 10061838 |
| Tamil Nadu | 77,165,000 | 6713355 | 0.169 | 1134557 | 12366671 | 3199451 |
| Uttar Pradesh | 238,875,000 | 20782125 | 0.565 | 11741901 | 127986717 | 33112160 |
| Uttarakhand | 11,795,000 | 1026165 | 0.321 | 329399 | 3590449 | 928905 |
| West Bengal | 99,723,000 | 8675901 | 0.331 | 2871723 | 31301783 | 8098260 |
| India (2015) | 1,430,000,000 | 124410000 | 0.403 | 50137230 | 549604315 | 70188422 |
| India (2021) * | 1,430,000,000 | 124410000 | 0.22 | 27370200 | 300032132 | 38316260 |

- **Estimates based on 2021 pneumonia incidence* [2]

# References

1. Wahl B, Knoll MD, Shet A, Gupta M, Kumar R, Liu L, et al. National, regional, and state-level pneumonia and severe pneumonia morbidity in children in India: modelled estimates for 2000 and 2015. Lancet Child Adolesc Heal. 2020;4:678–87.

2. National Health Mission. Guidance Note for Childhood Pneumonia Management. New Delhi; 2024. https://nhm.gov.in/New-Update-2024-26/SAANS 2024-25 Guidance Note_08112024.pdf.
